# HIFU-driven remodeling of fibrotic perivascular myeloid niches in hepatocellular carcinoma: implications for PD-1/PD-L1 blockade

**DOI:** 10.3389/fimmu.2026.2018159

**Published:** 2026-10-26

**Authors:** Zhen Wei, Yao Liao

**Affiliations:** 1 Department of Medical Equipment, Mianyang Central Hospital, School of Medicine, University of Electronic Science and Technology of China, Mianyang, China; 2 Sichuan Clinical Research Center for Radiation and Therapy, Mianyang Central Hospital, School of Medicine, University of Electronic Science and Technology of China, Mianyang, China; 3 Department of Oncology, Mianyang Central Hospital,School of Medicine, University of Electronic Science and Technology of China, Mianyang, China

**Keywords:** hepatocellular carcinoma, high-intensity focused ultrasound, liver fibrosis, macrophages, PD-1/PD-L1, perivascular niche

## Abstract

Hepatocellular carcinoma (HCC) usually develops within chronically inflamed and fibrotic liver tissue. This background creates an organ-specific immune niche in which macrophages, sinusoidal endothelial cells, activated stellate cells and extracellular matrix constrain T-cell access and sustain checkpoint-dependent tolerance. Spatial mapping places VEGFA-positive macrophages near CD34-positive endothelium and predicts VEGFA signaling, defining a candidate vascular barrier to PD-1/PD-L1 blockade. High-intensity focused ultrasound (HIFU) can disrupt tumor tissue and may alter this niche. Mechanical HIFU can fragment tumor tissue, release immunogenic material and activate dendritic cells, while focused ultrasound approaches can repolarize macrophages, increase T-cell infiltration and induce adaptive PD-L1 expression. These preclinical effects motivate combination with checkpoint inhibition, while direct niche remodeling in fibrotic HCC remains to be tested. This mini review examines how HIFU may remodel the fibrotic perivascular myeloid niche, why the resulting inflammatory window may sensitize HCC to PD-1/PD-L1 blockade, and which spatial and temporal biomarkers are required for clinical translation.

## 1 Introduction

Hepatocellular carcinoma (HCC) is the dominant form of primary liver cancer and commonly arises after years of chronic liver injury, inflammation and fibrosis (1, 2). Immune checkpoint inhibitors have changed systemic treatment, and atezolizumab plus bevacizumab established the clinical value of immune-based combinations in unresectable HCC (3). Nevertheless, response remains heterogeneous. The liver is a tolerogenic organ in which immune populations continuously interact with sinusoidal endothelial cells, hepatic stellate cells and extracellular matrix. During carcinogenesis, these components are reorganized into spatially restricted immunological niches that support immune exclusion and treatment resistance (4, 5). The inflammatory history of the liver is particularly relevant. Nonalcoholic steatohepatitis (NASH) can accumulate dysfunctional CD8+PD-1+CXCR6+ T cells and reduce the antitumor effect of PD-1 blockade in experimental HCC, showing that a checkpoint-positive phenotype does not necessarily indicate productive antitumor immunity (6). At the same time, spatial mapping of human HCC has identified intratumoral perivascular neighborhoods containing tissue-resident T cells, dendritic cells and macrophage subsets. VEGFA-positive perivascular macrophages lie near CD34-positive endothelial cells in these regions, with VEGFA-mediated signaling predicted by transcriptomic analysis (7). The problem is therefore not simply whether immune cells are present. Their position, activation state and vascular access determine whether they can participate in tumor control.

High-intensity focused ultrasound (HIFU) can perturb a local tumor region without systemic drug exposure. Depending on acoustic parameters, HIFU produces thermal coagulation or predominantly mechanical disruption, generating different debris, danger signals and immune consequences. A key question is whether HIFU can remodel the fibrotic perivascular myeloid niche sufficiently to make PD-1/PD-L1 blockade more effective. This has not been established in patients, but HCC biology and focused ultrasound models support a testable mechanistic framework.

## 2 Fibrotic perivascular myeloid niches as an organ-specific checkpoint barrier

### 2.1 Chronic liver injury establishes the niche

The normal hepatic sinusoid is specialized for immune tolerance. Liver sinusoidal endothelial cells can present antigen while maintaining low costimulation and inducible B7-H1/PD-L1, thereby promoting PD-1-dependent CD8+ T-cell tolerance (8). Chronic injury amplifies this program. Activated hepatic stellate cells deposit collagen and remodel vascular architecture, while macrophages are exposed to hypoxia, lipids and hepatocyte-derived danger signals. These pressures create a fibrotic compartment in which immunosuppressive myeloid cells and dysfunctional lymphocytes can persist (4, 5). Matrix deposition, vascular dysfunction and hypoxia may also shape antigen transport and immune-cell entry after focal injury. Fibrosis also changes the physical meaning of checkpoint inhibition. In fibrosis-associated HCC models, conventional PD-L1 antibody treatment can be less effective than a locally acting PD-L1 trap. The improved response of the trap was accompanied by reduced M2-like macrophages and polymorphonuclear myeloid-derived suppressor cells and by increased IFN-γ+TNF-α+ CD8+ T-cell infiltration (9). Human data likewise associate PD-L1 expression with macrophage infiltration and inflammatory activity in HCC (10). Thus, the PD-1/PD-L1 axis operates inside a stromal and myeloid context. Blocking the receptor-ligand interaction may be insufficient when the niche still limits antigen presentation, vascular trafficking and effector-cell positioning.

### 2.2 Perivascular macrophage-endothelial crosstalk and checkpoint resistance

Perivascular space is a logical control point because every circulating effector cell must cross or interact with the vascular interface before reaching tumor parenchyma. Human imaging mass cytometry shows that HCC contains distinct perivascular immune neighborhoods rather than a uniform immune infiltrate. These neighborhoods include tissue-resident T cells, dendritic cells and multiple macrophage populations. VEGFA-positive macrophages lie near CD34-positive endothelial cells, while transcriptomic analysis predicts VEGFA-mediated signaling (7). This adjacency motivates testing whether myeloid VEGFA signaling alters perfusion and CD8+ T-cell entry during checkpoint therapy. Resident Kupffer cells survey the sinusoids, whereas recruited monocyte-derived macrophages can acquire angiogenic or suppressive tumor-associated programs; neutrophils and myeloid-derived suppressor cells are distinct recruited populations (4, 5, 7). VEGFA staining alone does not assign the observed perivascular macrophages to either lineage.

Clinical anti-PD-1 studies support the importance of immune context. Molecular profiling of advanced HCC shows that response is enriched in tumors with pre-existing inflammatory and immune-active features rather than predicted by a single checkpoint marker (11). In neoadjuvant immunotherapy, high intratumoral tertiary lymphoid structure density is associated with pathologic response, and regressing regions retain T-cell and mature dendritic-cell interactions (12). Productive checkpoint therapy therefore depends on niche architecture as well as ligand expression.

## 3 How HIFU may remodel the perivascular niche

### 3.1 Mechanical versus thermal HIFU and antigenic material

HIFU has been used as a noninvasive ablative approach for liver tumors, including HCC in cirrhotic livers (13). Its immune effects depend on the mode of tissue destruction. Early HCC experiments showed that HIFU-generated debris could function as an antitumor vaccine in mice (14). Dendritic cells loaded with ultrasound-ablated tumor also induced tumor-specific immune responses *in vivo* (15). These studies established that local ultrasound injury can communicate with systemic immunity.

Mechanical HIFU provides a more direct bridge to niche remodeling. Compared with thermal HIFU, mechanical disruption generates fragmented RNA, DNA and protein material and can produce stronger dendritic-cell activation and T-cell priming under immune-stimulatory conditions (16). Mechanical ultrasound disruption has also been shown to modify the tumor microenvironment, repolarize tumor-associated macrophages toward an inflammatory state and increase T-cell infiltration. Importantly, adaptive PD-L1 expression appeared after treatment and anti-PD-L1 therapy amplified local and systemic antitumor effects (17). This sequence suggests a testable link to HCC vascular-myeloid organization, although the macrophage and T-cell measurements came from breast-tumor models (17).

Histotripsy adds HCC-model evidence. In bilateral subcutaneous models including Hepa1–6 HCC, partial histotripsy produced early HMGB1 release, innate activation, delayed CD8+ T-cell infiltration and inhibition of untreated tumors (18). A mechanical ultrasound needle likewise improved dendritic-cell presentation and synergized with anti-PD-L1 blockade (19). Perfluorohexane nanodroplet-assisted mechanical HIFU in subcutaneous HCC increased HMGB1, calreticulin and ATP, promoted dendritic-cell maturation and enhanced checkpoint therapy (20). In an orthotopic rat HCC model, partial histotripsy recruited CD11b-positive, NK and CD8+ cells over days 2–7, without modeling pre-existing fibrosis or checkpoint combination (21). **Table 1** separates these immune outcomes from the proposed perivascular mechanism.

**Table 1:** Evidence connecting the fibrotic perivascular myeloid niche with HIFU-responsive immune remodeling.

Evidence domain	Model/setting	Key observation	Implication for HIFU-PD-1/PD-L1 combination	Reference
Perivascular HCC niche	16 resected human HCCs; VEGFA proximity analysis in 8	Perivascular neighborhoods contained tissue-resident T cells, dendritic cells and macrophages; VEGFA-positive macrophages lay near CD34-positive endothelium, with predicted VEGFA signaling.	Defines a spatial target for measuring vascular access and myeloid state before and after HIFU.	(7)
Fibrotic checkpoint niche	Two fibrosis-associated murine HCC models plus human single-cell datasets	PD-L1 trap reduced marker-defined M2-like macrophages/PMN-MDSCs and increased IFN-γ+TNF-α+ CD8+ T cells.	Links checkpoint efficacy to remodeling of the fibrotic myeloid compartment.	(9)
Mechanical HIFU plus ICI	Murine breast-tumor models	Mechanical disruption increased inflammatory macrophage features and T-cell infiltration; delayed PD-L1 induction enabled anti-PD-L1 synergy.	Supports an inflammatory, checkpoint-limited state in breast-tumor models.	(17)
Histotripsy in HCC	Bilateral subcutaneous Hepa1–6 HCC model	Early HMGB1/innate activation was followed by CD8+ infiltration and inhibition of untreated tumors.	Shows local and systemic immune effects without testing fibrosis or perivascular conversion.	(18)
Mechanical HIFU in liver cancer	Subcutaneous HCC with perfluorohexane nanodroplets	HMGB1, calreticulin and ATP increased with dendritic-cell maturation, T-cell activation and improved checkpoint therapy.	Subcutaneous HCC-model evidence for mechanically enhanced HIFU sensitization.	(20)

### 3.2 Myeloid repolarization, endothelial access and T-cell entry

A testable niche-level effect of HIFU is a change in macrophage function and spatial relationships. Focused ultrasound can generate inflammatory cytokines and danger-associated molecular patterns that recruit antigen-presenting cells. Histotripsy studies emphasize innate activation and systemic immune propagation (22). In poorly immunogenic tumors, boiling histotripsy plus *in situ* CD40 stimulation increased a marker-defined M1:M2 macrophage ratio, functional CD8+ T cells and checkpoint-blockade efficacy (23). This ratio describes polarization markers, not a fixed functional state; antigen presentation, cytokine output, VEGFA/TGF-β signaling and support of CD8+ cells require direct assays. Fibrotic matrix, activated stellate cells, impaired perfusion and hypoxia may alter focal tissue injury and subsequent immune-cell movement. Histotripsy susceptibility depends on tissue mechanics; major liver vessels and fibrillar collagen can persist after fractionation in non-tumor experiments (24, 25). Conversely, boiling histotripsy reduced collagen measures in a non-tumor liver-fibrosis model (26). Collagen continuity, stellate-cell activity, hypoxia and perfusion therefore need measurement alongside immune entry in fibrotic HCC: antigen release alone does not establish a traversable vascular-stromal barrier. Ultrasound-targeted microbubble destruction can modulate vascular trafficking (27), but its tissue effect differs from HIFU.

As shown in **Figure 1**, a conservative model contains three linked compartments. The baseline fibrotic HCC niche includes activated stellate cells and extracellular matrix, VEGFA-positive perivascular macrophages, tolerogenic endothelium and PD-L1-dependent T-cell restraint. HIFU then creates a local injury field that releases antigen and damage signals; downstream myeloid and vascular changes are the proposed HCC-specific link. The third compartment represents checkpoint blockade acting after immune recruitment has begun. The macrophage redistribution, restored vascular access and durable CD8+ T-cell entry illustrated in **Figure 1** remain hypotheses for fibrotic HCC.

**Figure 1:**
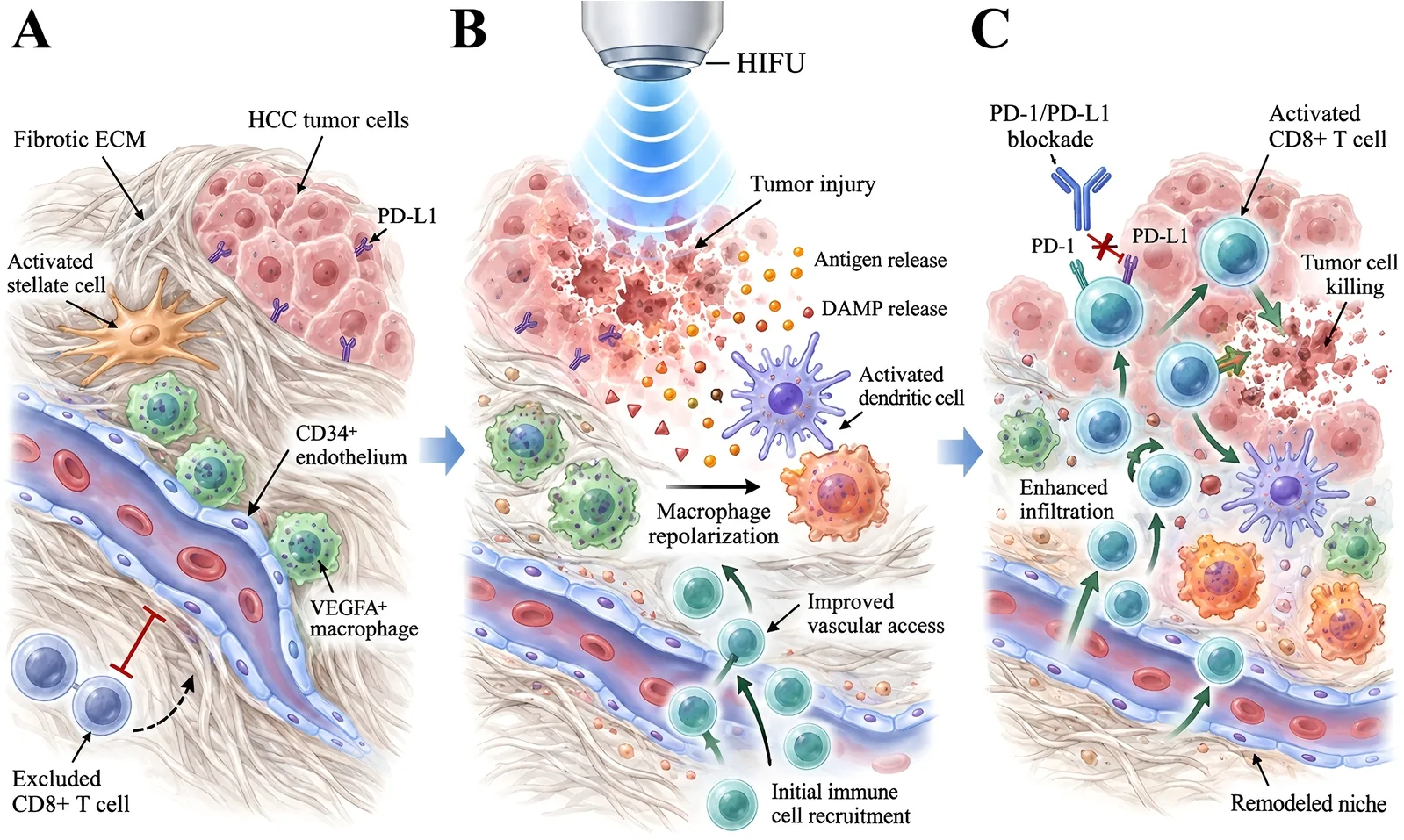
HIFU-mediated remodeling of the fibrotic perivascular immune niche in hepatocellular carcinoma. Conceptual schematic: **(A)** draws on human HCC spatial mapping; **(B)** antigen release has preclinical support. All arrows or labels suggesting macrophage repolarization, restored vascular access, CD8+ entry or **(C)** tumor control in fibrotic HCC depict hypotheses.

## 4 Why PD-1/PD-L1 blockade is a rational second hit

### 4.1 Adaptive checkpoint induction after focused ultrasound

Local inflammation creates both opportunity and negative feedback. Immunogenic cell death can improve antigen presentation and T-cell priming, but activated T cells release IFN-γ and other cytokines that can drive adaptive PD-L1 expression in tumor and myeloid cells. This is one reason why ablation alone often produces incomplete systemic immunity (28). In the mechanical HIFU study, PD-L1 increased on several infiltrating immune populations after treatment, and combination with anti-PD-L1 improved control of treated and distant lesions (17). The checkpoint signal was therefore not merely a pre-existing biomarker. It emerged as part of the inflammatory response. Histotripsy also induced transient CD11b-positive and Ly6G-positive influx in flank models (18), without establishing suppressive function for those cells. A later repair phase could recruit suppressive monocytes, neutrophils or myeloid-derived suppressor cells and re-engage hypoxia, VEGFA and TGF-β signaling, limiting sustained CD8+ function even when infiltration rises.

This temporal behavior is particularly relevant in HCC, where PD-L1 may already be expressed by macrophages in fibrotic tissue (9, 10). HIFU could increase the number of activated effector cells while simultaneously strengthening a PD-1/PD-L1 brake. Blocking that brake during the priming-to-effector transition may preserve the inflammatory gain created by ultrasound. The logic differs from giving two independent antitumor treatments. HIFU may initiate immune activity, while checkpoint blockade can sustain T-cell function during adaptive PD-L1 induction; later stromal or myeloid repair remains a separate determinant of response.

### 4.2 Timing and sequence of combination treatment

The optimal sequence is not known. Immediate checkpoint blockade may be useful when inhibitory signaling is already dominant, but it could precede adequate antigen release and dendritic-cell maturation. Delayed treatment may capture adaptive PD-L1 induction but miss an early window of T-cell priming. Preclinical studies place danger signals and innate-cell influx in the early hours to days, followed by dendritic-cell presentation, CD8+ expansion and adaptivePD-L1 expression over subsequent, model-dependent intervals (16–18, 21). Thermal versus mechanical injury, treated tumor fraction and burden, baseline immune state, and liver etiology or fibrosis may shift these phases; schedules should follow serial biological readouts.

A translational design can sample the first 72 hours for HMGB1, calreticulin, inflammatory monocyte/neutrophil influx and dendritic-cell uptake, then subsequent days for antigen presentation, CD8+ expansion and function, perfusion, macrophage state and PD-L1 induction. Later measurements should track suppressive myeloid subsets, hypoxia, VEGFA/TGF-β signaling and persistent CD8+ activity. Pretreatment, concurrent and biomarker-timed blockade arms can then be compared within each acoustic modality and liver-disease stratum (16, 17, 29). **Figure 2** depicts a candidate sequence rather than a fixed treatment window.

**Figure 2:**
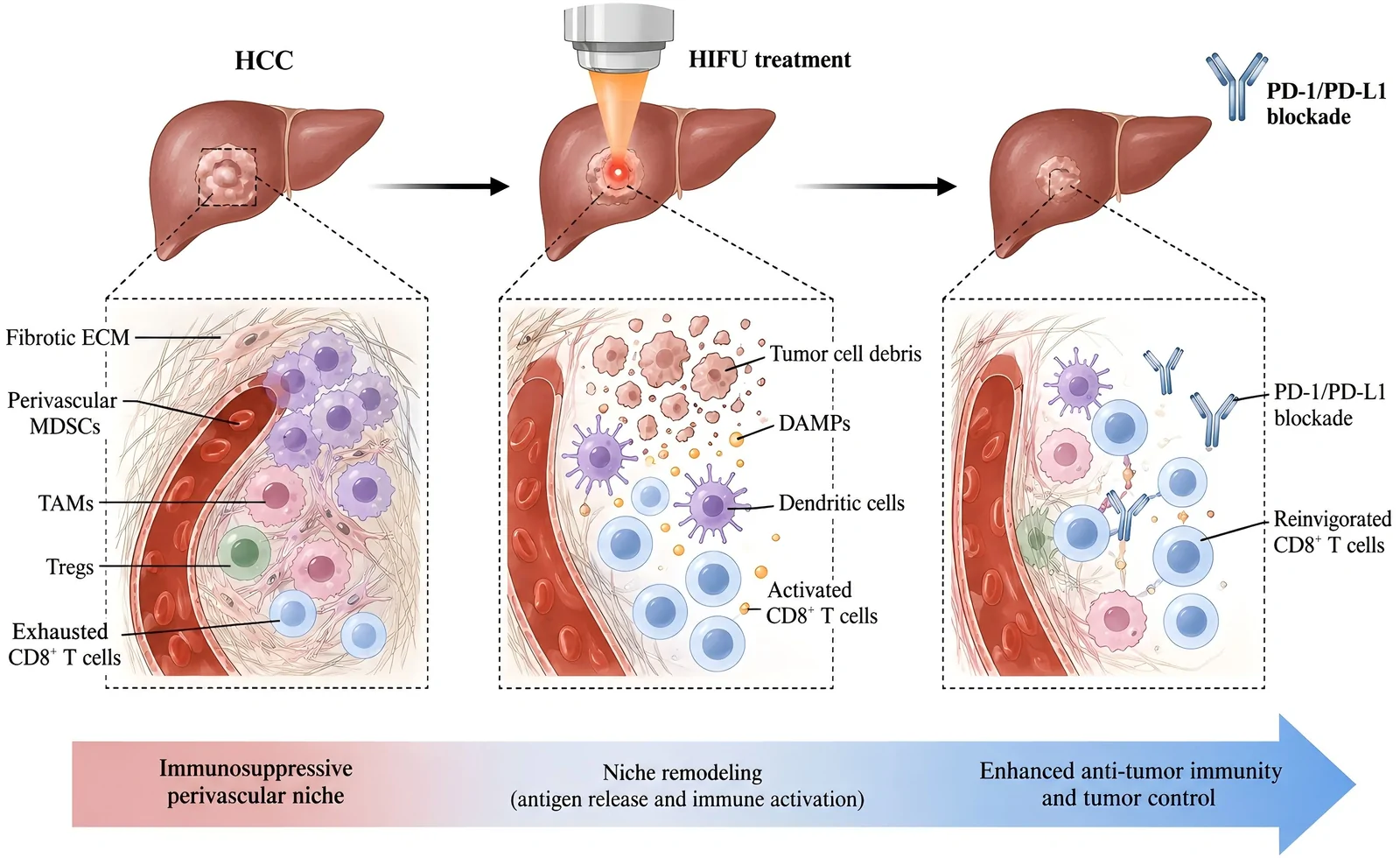
Sequential immune remodeling by HIFU and PD-1/PD-L1 blockade in hepatocellular carcinoma. The depicted niche remodeling and tumor control in fibrotic HCC are hypotheses. Timing and persistence may differ by modality, tumor burden and liver disease.

## 5 Translational framework for fibrotic HCC

### 5.1 Niche-resolved biomarkers

Tumor shrinkage cannot show whether the perivascular niche was remodeled. Baseline biopsies should document collagen, activated stellate cells, hypoxia/perfusion, lineage-aware macrophage phenotypes, endothelial features and CD8+ T-cell distance from vessels and tumor cells. Post-HIFU biopsies, when feasible, should repeat these spatial measurements and assess matrix continuity. Imaging mass cytometry or multiplex immunofluorescence can quantify VEGFA-positive macrophage proximity to CD34-positive endothelium and map dendritic cells and CD8+ T cells (7). Origin-aware single-cell markers can separate Kupffer-like from recruited monocyte-derived populations, with lineage and function tested experimentally. Blood biomarkers are less specific but can support temporal interpretation. Circulating cytokines, soluble checkpoint molecules and peripheral T-cell activation may indicate systemic immune propagation. Functional imaging could be integrated with perfusion assessment to determine whether acoustic treatment changed vascular accessibility. The key endpoint is a coordinated niche transition: reduced suppressive myeloid activity, increased antigen presentation, sustained CD8+ T-cell function and improved proximity to tumor cells and a checkpoint signal that is pharmacologically targetable. As shown in **Table 2**, these measurements convert a broad HIFU-plus-immunotherapy concept into a sequence of testable biological decisions.

**Table 2:** Niche-resolved translational framework for sequencing HIFU with PD-1/PD-L1 blockade in fibrotic HCC.

Decision point	Biological rationale	Suggested readout	Interpretation	Supporting reference
Baseline niche	Fibrosis and etiology alter macrophage, endothelial and T-cell states.	Fibrosis/collagen and etiology; hypoxia/perfusion; Kupffer-like versus recruited myeloid markers; VEGFA-positive macrophage/CD34-positive vessel proximity; CD8+ distance to vessels.	Classifies macrophage-rich immune exclusion versus pre-existing immune organization.	(6, 7, 9)
HIFU mode and immediate injury	Mechanical and thermal HIFU generate different debris and immune contexts.	Ablation geometry and matrix integrity; perfusion; HMGB1; calreticulin; ATP.	Confirms immunogenic local injury rather than only coagulative destruction.	(16, 20)
Early myeloid transition	DAMPs and antigen uptake should precede productive T-cell entry.	Activated dendritic cells; lineage-resolved macrophage location and antigen-presentation/cytokine functions; VEGFA/TGF-β.	Distinguishes marker shifts from functional and spatial niche change.	(17, 23)
Adaptive checkpoint phase	IFN-driven inflammation can induce PD-L1 after ultrasound treatment.	Serial tumor/myeloid PD-L1, PD-1 and IFN-γ; CD8+ function and vessel-to-tumor distance; late suppressive myeloid influx, hypoxia and TGF-β.	Tests blockade timing against adaptive signaling and suppressive rebound.	(9, 17)
Systemic conversion	Local remodeling should propagate beyond the treated acoustic field if the combination generates systemic immunity.	Untreated-lesion biopsy/imaging; tumor-reactive T-cell clonotypes; distant CD8+ infiltration.	Separates local ablation from systemic immune sensitization.	(18, 23)

### 5.2 Fibrotic-liver safety and patient selection

Patient selection must consider the diseased liver rather than the tumor alone. HIFU is noninvasive, but fibrosis, tumor position, acoustic window, adjacent bowel and vascular structures influence feasibility. Focal injury geometry and perfusion should be measured across fibrosis strata rather than inferred from delivered acoustic settings. Comparative ablation literature emphasizes that treatment choice depends on tumor size, location and liver reserve (30–32). The immunological objective should not justify unsafe energy delivery or excessive loss of functional parenchyma.

Etiology may also alter benefit. The NASH-associated immune state described with PD-1 therapy indicates that an inflamed liver can contain activated yet nonproductive T cells (6). In this context, stronger inflammation is not automatically desirable. Patients with severe steatohepatitis or active inflammatory injury may require different acoustic dosing and closer monitoring of liver function. Conversely, tumors with macrophage-rich, immune-excluded perivascular niches may be more suitable for a remodeling strategy than tumors already containing organized lymphoid structures and abundant effector-cell access (12). A niche-based trial should therefore stratify by fibrosis, etiology and baseline spatial immune architecture rather than treating all HCC as one immunological state.

## 6 Challenges and research priorities

Three gaps limit translation. First, existing HIFU and histotripsy studies show antigen release, myeloid changes and T-cell recruitment, but do not prove selective remodeling of VEGFA-positive perivascular macrophage-endothelial neighborhoods in fibrotic human HCC. Paired spatial biopsies should quantify vessel-adjacent macrophages, endothelial contacts, vessel distribution and perfusion, and CD8+ vessel-to-tumor migration. A fibrosis-associated orthotopic HCC model could combine these measurements with lineage-restricted macrophage perturbation or myeloid VEGFA-pathway modulation; a treatment-by-perturbation interaction on checkpoint efficacy would test the niche’s causal role. Second, thermal HIFU, mechanical HIFU, boiling histotripsy, cavitation-enhanced HIFU and ultrasound-targeted microbubble destruction are not interchangeable. Immune phenotypes should be linked to measured acoustic dose, matrix integrity, vascular function and tissue effect.

Third, the field needs a schedule-defining biomarker. Serial sampling should determine whether DAMP release precedes dendritic-cell activation, whether macrophage repolarization precedes CD8+ T-cell entry, and when PD-L1 is induced and whether later myeloid, hypoxic or TGF-β-associated repair curtails CD8+ function. A small mechanistic trial with paired biopsies may be more informative than a large uncontrolled combination cohort. Concepts of immunogenic cell death and focused ultrasound immunotherapy provide the framework (29, 33, 34). Future work should also distinguish local control from systemic immune conversion. A successful HIFU procedure can destroy the treated lesion without remodeling untreated sites. Abscopal inhibition after histotripsy and enhanced distant control with checkpoint combinations indicate that systemic effects are possible, but they remain inconsistent (18, 23). The strongest translational endpoint would therefore combine local spatial niche remodeling with evidence of clonally related T-cell expansion or immune change in an untreated lesion. Such data would show that local disruption of an organ-specific niche can propagate beyond the acoustic field.

## 7 Conclusion

Fibrotic HCC contains an organ-specific immunological architecture in which macrophages, sinusoidal endothelium, stromal fibrosis and checkpoint signaling converge around vascular access. Spatial evidence identifies perivascular immune neighborhoods as a discrete unit of this architecture. HIFU can release tumor antigens and danger signals, activate dendritic cells, alter macrophage states and promote T-cell infiltration. These preclinical effects justify testing focused ultrasound with PD-1/PD-L1 blockade when treatment-induced inflammation creates adaptive checkpoint expression, while tracking matrix and myeloid repair.

The central uncertainty is spatial. It is not yet known whether HIFU can reproducibly dismantle or reprogram the fibrotic perivascular myeloid niche in human HCC. Addressing this gap requires paired niche-resolved sampling, acoustic-mode-specific reporting and biologically timed checkpoint therapy. If these elements are incorporated into early clinical studies, the combination can be evaluated as a defined sequence of niche remodeling and checkpoint release rather than as an empirical pairing of local ablation with systemic immunotherapy.
